# Measurement and Control of Radially Polarized THz Radiation from DC-Biased Laser Plasma Filaments in Air

**DOI:** 10.3390/s22145231

**Published:** 2022-07-13

**Authors:** Bonan Han, Yanping Chen, Tianhao Xia, Linzheng Wang, Chen Wang, Zhengming Sheng

**Affiliations:** 1 Key Laboratory for Laser Plasmas (Ministry of Education), School of Physics and Astronomy, Shanghai Jiao Tong University, Shanghai 200240, China; hbn0528@sjtu.edu.cn (B.H.); xth1996@sjtu.edu.cn (T.X.); linzhengwangsjtu@sjtu.edu.cn (L.W.); w_chen@sjtu.edu.cn (C.W.); zmsheng@sjtu.edu.cn (Z.S.); 2 Collaborative Innovation Center of IFSA, Shanghai Jiao Tong University, Shanghai 200240, China; 3 Tsung-Dao Lee Institute, Shanghai Jiao Tong University, Shanghai 200240, China

**Keywords:** terahertz radiation, laser plasmas, radial polarization, longitudinal dc-biased electric field

## Abstract

Detection and manipulation of radially polarized terahertz (THz) radiation is essential for many applications. A new measurement scheme is proposed for the diagnosis of radially polarized THz radiation from a longitudinal dc-biased plasma filament, by introducing a movable metal mask. The amplitude and spectrum of the radially polarized THz beam was measured with a <110>-cut ZnTe crystal, where the THz beam pattern was modulated by the mask. Based on this measurement scheme, it was demonstrated that the amplitude and spectrum of the radially polarized THz radiation from the longitudinal dc-biased filament could be manipulated by controlling the strength and the location of the dc-biased field.

## 1. Introduction

Radially polarized terahertz (THz) radiation is a special THz vector beam whose polarization direction is along the radial direction in the beam cross section [1]. Longitudinal THz electric fields can be formed by tightly focusing radially polarized terahertz radiation, which have important applications in electron acceleration [2], optical tweezers [3], THz imaging [4], etc. In recent years, many research groups have proposed a variety of methods to generate radially polarized THz radiation. Ryo et al. generated radially and azimuthally polarized THz beams by piecing together nonlinear crystals [5]. Cliffe et al. used a radially biased photoconductive antenna to generate a longitudinal THz electric field up to 2 kV/cm after focusing [6]. Using segmented waveplates, linearly polarized THz radiation can be converted to radially polarized THz radiation [2,7]. Additionally, strong radially polarized broadband THz radiation can be generated in accelerator-based light sources through coherent diffraction and transition radiation [8,9]. D’Amico et al. generated radially polarized THz radiation from laser plasmas [10]. Later, Liu et al. increased the intensity of this THz radiation by an order of magnitude by introducing an external electric field to laser plasmas [11].

Recently, THz radiation from laser plasmas has attracted broad interest due to its high damage threshold and ultrabroad spectral bandwidth, compared with other methods [12,13,14,15,16,17,18]. However, effective characterization of the radially polarized THz radiation from laser plasmas is still challenging. Heterodyne detectors have been used for the measurement of radially polarized THz at specific frequencies [10,11,18]. Rizaev et al. discussed the spectral distributions of radially polarized THz radiation from DC-biased laser plasmas by using a bolometer with THz filters [16]. Fukuda et al. measured the angular distribution of radially polarized THz by calibrated diode detectors with sensitive bands at 0.14 THz to 0.33 THz [14]. The above methods can obtain the intensity and spatial distribution of the radially polarized terahertz wave, but the electric field and the corresponding spectrum of this THz signal cannot be obtained. The waveform of the longitudinal THz electric field formed by focusing the radially polarized THz emission has been demonstrated by using <100>-cut GaP or ZnTe crystal [16,19], whose signal-to-noise ratio is much lower than the measurement of the linearly polarized THz signal with a <110>-cut ZnTe crystal. Following this, some research groups divided the emitted radially polarized THz beam into four pieces with a sectorial mask, and measured the waveform of this THz signal piece-by-piece with a <110>-cut ZnTe crystal [19,20]. However, with this method, alignment is not easy because the resultant THz signal is very sensitive to the transverse position of the sectorial mask with respect to the THz beam profile.

In this paper, a new method is proposed for the measurement of radially polarized THz radiation from a plasma filament with a longitudinally oriented external electric field. The waveform and corresponding spectrum of the radially polarized THz pulse from a plasma filament can be distinguished and obtained by electro–optic (EO) sampling technique with a <110>-cut ZnTe crystal, by modulating the THz beam pattern with a movable metal mask. Based on this measurement scheme, it will be demonstrated that the amplitude and spectrum of the radially polarized THz radiation from a longitudinally dc-biased filament can be manipulated by control of the amplitude and the location of the external electric field, respectively. This paper is organized as follows. In Section 2, the detection method for the radially polarized THz radiation is presented. In Section 3, the experimental result based on the measurement scheme is shown. Based on the experimental result, the THz spatial distribution is analyzed by the transition-Cherenkov radiation principle. In Section 4, the radially polarized THz radiation is manipulated by adjusting the external electric field. Finally, a summary is given in Section 5.

## 2. Detection Methods

**Figure 1:**
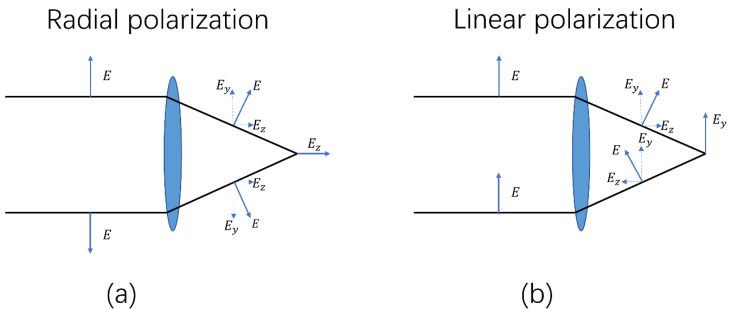
The directions of the electric fields at the focal plane originating from (**a**) radially polarized THz waves, and (**b**) linearly polarized THz waves.

THz radiation from plasma filaments with an external electric field can be either radially polarized (with a longitudinally oriented dc-bias) [11] or linearly polarized (with a transversely-oriented dc-bias) [21]. When the THz radiation is collected and focused, the polarization of the radially polarized THz components will become longitudinal at the focal plane (Figure 1a), while the polarization of the linearly polarized THz components is in the transverse direction at the focal plane (Figure 1b). Detection of the linearly polarized THz radiation often uses an electro–optic sampling technique with a <110>-cut ZnTe crystal [22]. For a longitudinal THz electric field, however, it will not change the refractive index of the ZnTe crystal in the (110) plane. Therefore, the longitudinal THz components at the focal plane (as *E_z_* in Figure 1a), which originated from the radially polarized THz components, cannot be directly measured by electro–optic sampling with a <110>-cut ZnTe crystal, for normal incidence.

**Figure 2:**
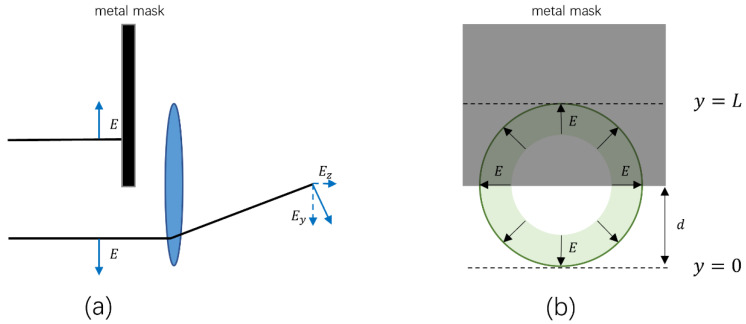
Side view (**a**), and front view (**b**), of the schematic diagram for beam shielding with a rectangular metal mask.

To measure the waveforms of the radially polarized and linearly polarized THz radiation from plasma filaments at the same time, we introduced a rectangular metal plate in front of the focusing optics as a mask to block part of the THz radiation, as shown in Figure 2. As the lower edge of the mask moves from the top to the bottom of the THz beam pattern, the residual THz radiation with radial polarization will occur transversely polarized THz components, *E_y_*, at the focal plane (as *E_y_* in Figure 2a) while the residual THz radiation with linear polarization retains its polarization. Thus, the waveforms of the radially polarized THz radiation can be obtained by measuring the THz components, *E_y_*, at the focal plane with a metal mask and a <110>-cut ZnTe crystal.

**Figure 3:**
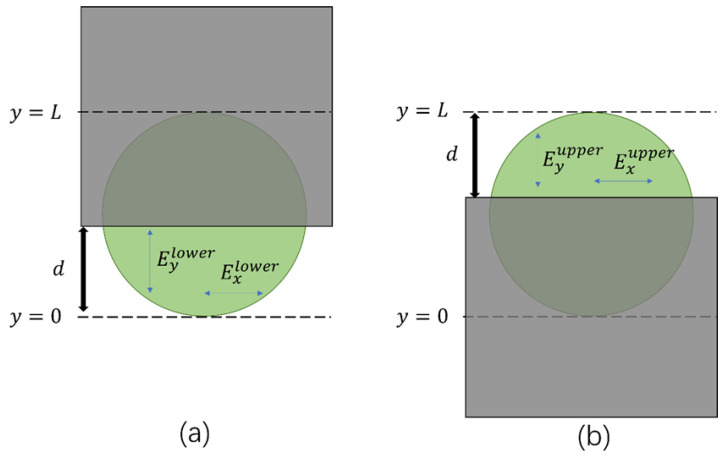
Determination the boundary of the THz beam pattern by moving a metal mask upwards (**a**), and downwards (**b**).

To determine the spatial distribution of the THz radiation from plasma filaments, we first placed the metal mask so as to completely block the THz beam pattern. Then the mask was moved upwards along the *y*-axis until the THz signal started to appear. This particular location of the lower edge of the mask was defined as *y* = 0, corresponding to a lower edge of the THz beam pattern, as shown in Figure 3a. As the mask was moved further upwards, the distance between the lower edge of the mask and the location *y* = 0 was defined as *d*, which had close correlation to the THz signal arriving at the detector. When the THz signal remained constant while the mask was moved upwards, the location of the lower edge of the mask was defined as *y* = *L* (corresponding to upper edge of the THz beam pattern). Thus, we could roughly determine the boundary of the THz beam pattern by scanning the metal mask across the whole THz beam profile in *y* direction. In the measurement, we could obtain the horizontally polarized and the vertically polarized THz electric fields at the focal plane by rotating the angles of a half-wave plate (HWP) and a ZnTe crystal in the electro–optic sampling system [21,23]. Here, we defined the horizontally and vertically polarized THz signals as $E_{x}^{lower}$ and $E_{y}^{lower}$ (Figure 3a), respectively. When *d* was changed from 0 to *L*, the horizontal and the vertical components of the linearly polarized THz signal became larger with the increase in *d*. For the radially polarized THz beam, however, its vertical components increased from zero to a maximum, when *d* changed from 0 to *L*/2. For *d* larger than *L*/2, the THz signals from the upper half of the radially polarized THz beam (with inversed polarization) canceled the THz signals from the lower half, leading to a decrease in total THz vertical components at the focal plane. Meanwhile, the horizontal THz components from a radially polarized THz beam remained at zero when moving the mask vertically (along the *y*-axis) because the horizontal THz components from the left half and the right half of this THz beam canceled each other at the focal plane. In order to improve the accuracy of our experiment, being certain that radially polarized THz radiation had been measured, we also measured the THz radiation by moving the metal mask in the opposite direction, as shown in Figure 3b. After blocking the whole THz beam pattern with the mask, we slowly moved the mask downwards. In the same way, the boundary of the THz beam pattern could be decided when the upper edge of the mask was located at *z* = *L* (corresponding to upper edge of the THz beam pattern) and *z* = 0 (corresponding to lower edge of the THz beam pattern). In this case, *d* was defined as the distance between the upper edge of the THz beam pattern and the upper edge of the mask, while the horizontally polarized and the vertically polarized THz signals were defined as $E_{x}^{upper}$ and $E_{y}^{upper}$, respectively.

## 3. Results

**Figure 4:**
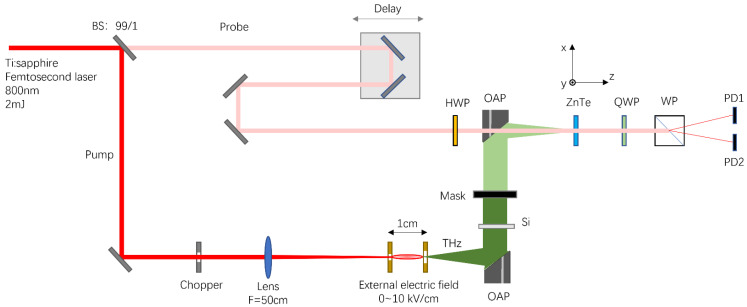
Schematic diagram for the experimental setup: HWP, half-wave plate; QWP, quarter-wave plate; WP, Wollaston prism; PD, photodiode detector.

The experimental setup for the generation and the detection of the THz radiation from laser-induced air plasmas is sketched in Figure 4. An 800 nm, 40 fs, 2 mJ laser was divided by a beam splitter (BS) into a pump beam and a probe beam. The pump beam was focused by a convex lens with 50 cm focal length to produce a 1 cm long laser filament in air. Two copper electrodes (with a hole in the center of each copper plate) with a diameter of 5 cm were set on both sides of the filament, forming a longitudinally oriented external electric field along the filament. The distance between the two copper plates was around 1 cm and the voltage applied to the plates could be varied from 0 to 10 kV. Thereafter, THz radiation from the longitudinally dc-biased filament was collected by two off-axis parabolic mirrors (OAP) with focal length of 10 cm and measured by electro–optic sampling technique with a <110>-cut ZnTe crystal. The diameters of the off-axis parabolic mirrors were both 50 mm, and they could collect THz radiation at an angle of up to 15 degrees from axis z. A silicon wafer was placed between the two off-axis parabolic mirrors to separate the THz beam from the pump beam. A rectangular metal mask, which could be moved along the *y*-axis, was placed just after the silicon wafer to control the THz beam pattern that reached the detector.

**Figure 5:**
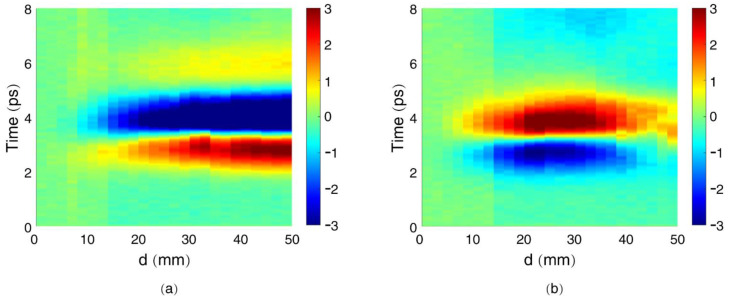
Measured THz radiation from a dc-biased filament with a longitudinal external electric field along the filament: (**a**) horizontally polarized component ($E_{x}^{lower}$); (**b**) vertically polarized component ($E_{y}^{lower}$).

Figure 5 shows the evolution of the measured THz waveforms when scanning the metal mask upwards (along the *y*-axis). $E_{x}^{lower}$ and $E_{y}^{lower}$ correspond to the measured THz waveforms of the horizontally polarized THz components and the vertically polarized THz components when moving the metal mask downwards (Figure 5a,b), respectively. Each vertical line relates to a THz waveform obtained at a specified *d*. The signals with d=0 were obtained when the THz radiation was completely blocked by the metal mask. The radially polarized THz beam was centrosymmetric about the center of the THz beam pattern, so the left half and the right half of the THz beam canceled each other in the horizontal polarization at the focal plane when the mask was moved along the vertical direction (parallel to the *y*-axis). As a result, the radially polarized THz radiation did not contribute to the measured horizontally polarized THz components, $E_{x}^{lower}$. As the external electric field along the filament was not perfectly longitudinally oriented, the measured $E_{x}^{lower}$ corresponded to the horizontal components of the linearly polarized THz radiation from the filament with a transversely oriented external electric field [21]. In this case, the measured $E_{x}^{lower}$ increased with an increase in *d*, as shown in Figure 5a. As for the vertically polarized THz components, $E_{y}^{lower}$, however, the measured waveforms were contributed by both the radially polarized THz radiation and the linearly polarized THz radiation. As the polarity of the radially polarized THz beam was opposite between the upper and lower halves, the measured vertically polarized THz components, $E_{y}^{lower}$, had a decreasing tendency at d>25 mm, as shown in Figure 5b. When the mask was completely removed (*d* = 50 mm in Figure 5b), the measured $E_{y}^{lower}$ only corresponded to the vertical components of the linearly polarized THz radiation from the filament with a transversely oriented external electric field, because the THz signals from the upper half of the radially polarized THz beam canceled those from the lower half.

As mentioned above, not only a radially polarized THz beam, but also a linearly polarized THz beam were generated in our experiment when applying a longitudinally oriented dc-bias to the filament. This was due to a slight deviation in the direction the dc-bias with respect to the propagation direction of the laser beam, which induced a transverse component of the external electric field to the plasma filament responsible for the generation of the linearly polarized THz beam [22]. We will now discuss how to distinguish the THz signals for the radially polarized THz beam from those for the linearly polarized THz beam. In Figure 5b, the measured THz signal $E_{y}^{lower}$ involves vertical components from both a linearly polarized THz beam and a radially polarized THz beam. In order to obtain the radially polarized THz signal, we need to subtract the linearly polarized THz component from $E_{y}^{lower}$. The measured THz signal $E_{x}^{lower}$ only involves horizontal components from a linearly polarized THz beam. Thus, from the measured horizontal THz components in Figure 5a, we can obtain the ratio between the THz electric field obtained with the mask located at *d* $E_{x}^{lower}\left(d\right)$ and the THz electric field obtained without the mask (located at *L* = 50 mm) $E_{x}^{lower}\left(L\right)$ as:

(1) $$\alpha^{lower}\left(d\right)=E_{x}^{lower}\left(d\right)/E_{x}^{lower}\left(L\right).$$

For the linearly polarized THz beam, the vertical THz components should have the same relation to the mask location *d*. Therefore, it can be derived that:

(2) $$\alpha^{lower}\left(d\right)=E_{lin}^{lower}\left(d\right)/E_{lin}^{lower}\left(L\right),$$

where $E_{lin}^{lower}\left(d\right)$ is the electric field of the linearly polarized THz beam when the mask is located at position *d*. In Figure 5b, the measured THz signal $E_{y}^{lower}\left(L\right)$ only corresponds to the vertical components of the linearly polarized THz beam, as the vertical components from the upper half and lower half of the radially polarized THz beam cancel each other. Thus, the vertical components of the linearly polarized THz beam with the mask located at *d*, denoted as $E_{y,lin}^{lower}\left(d\right)$, can be derived as:

(3) $$E_{y,lin}^{lower}\left(d\right)=E_{y}^{lower}\left(L\right)\cdot \alpha^{lower}\left(d\right).$$

and then, the vertical components of the radially polarized THz beam, denoted as $E_{y,rad}^{lower}\left(d\right)$, can be derived as:

(4) $$\begin{array}{c} E_{y,rad}^{lower}\left(d\right)=E_{y}^{lower}\left(d\right)-E_{y,lin}^{lower}\left(d\right)\\ =E_{y}^{lower}\left(d\right)-E_{y}^{lower}\left(L\right)\cdot E_{x}^{lower}\left(d\right)/E_{x}^{lower}\left(L\right). \end{array}$$

In this way, we can obtain the waveform and the one dimensional spatial distribution of the vertical components of the radially polarized THz beam. In the same way, the radially polarized THz signal can be obtained by scanning the mask downwards as:

(5) $$E_{y,rad}^{upper}\left(d\right)=E_{y}^{upper}\left(d\right)-E_{y}^{upper}\left(L\right)\cdot E_{x}^{upper}\left(d\right)/E_{x}^{upper}\left(L\right).$$

**Figure 6:**
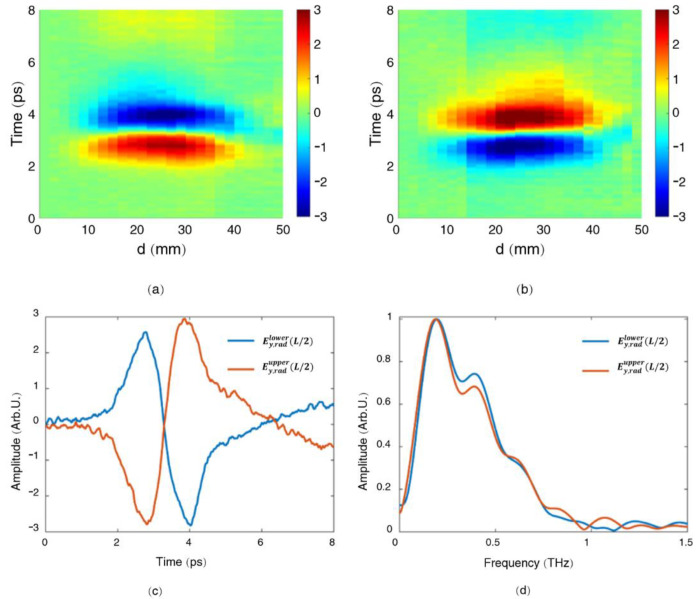
Vertical components of the radially polarized THz beam when scanning the mask upwards ($E_{y,rad}^{lower}\left(d\right)$) (**a**), and downwards ($E_{y,rad}^{upper}\left(d\right)$) (**b**); (**c**,**d**) are, respectively, the waveforms and the corresponding spectra of $E_{y,rad}^{lower}\left(L/2\right)$ and $E_{y,rad}^{upper}\left(L/2\right)$.

Based on Equations (1)–(5), the vertical components of the radially polarized THz beam when scanning the mask upwards and downwards, are shown in Figure 6a,b, respectively. The measured radially polarized THz signal reaches its maximum when the mask blocks exactly half of the THz beam pattern ($E_{y,rad}^{lower}\left(L/2\right)$ and $E_{y,rad}^{upper}\left(L/2\right)$), while it goes back to zero when the mask is completely removed ($E_{y,rad}^{lower}\left(L\right)$ and $E_{y,rad}^{upper}\left(L\right)$). The waveform of the vertical components from the upper half of the radially polarized THz beam $E_{y,rad}^{upper}\left(L/2\right)$ has opposite polarity compared with that from the lower half of the radially polarized THz beam $E_{y,rad}^{lower}\left(L/2\right)$ (as shown in Figure 6c) while their corresponding spectra remain the same (as shown in Figure 6d). All these behaviors match well with the characteristic of a radially polarized THz beam. Therefore, based on our method, the signal for a radially polarized THz beam can be well distinguished from the measured THz signals with other polarizations (a linear polarization in this experiment).

**Figure 7:**
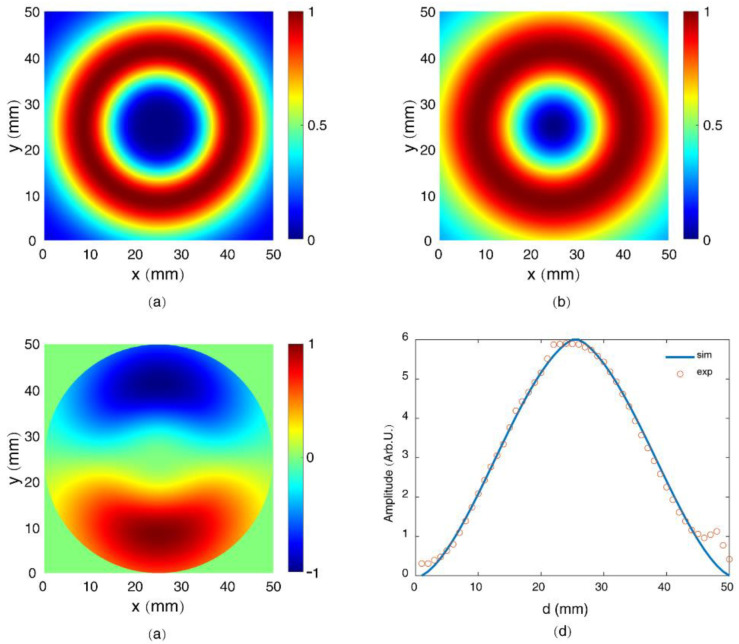
Spatial distribution of (**a**) the energy density, (**b**) the field amplitude, and (**c**) the vertical components of the THz radiation from a longitudinally dc-biased filament. (**d**) Simulation and experimental results for THz amplitude as a function of *d*.

Based on the measurement, we can also analyze the spatial distribution of the radially polarized THz radiation from the longitudinally dc-biased filament. A focused femtosecond laser will induce plasmas in air and form a long laser filament due to an interplay between the Kerr-focusing effect and the plasma-defocusing effect. Inside the filament, the laser-induced ponderomotive force will drive the electrons to produce longitudinal oscillations, which can be regarded as a dipole-like charge current, $j_{z}^{w}\left(\omega \right)$, oriented along the filament [10]. When the filament is applied by an external electric field, $E_{ext}$, with its orientation parallel to the filament, the electrons ionized by the laser will also be driven by the external electric field to form a current, $j_{z}^{e}\left(\omega \right)$, which is proportional to the amplitude of the external electric field [11]. Consequently, the total longitudinal electron current can be expressed as $j_{z}\left(\omega \right)=j_{z}^{w}\left(\omega \right)+j_{z}^{e}\left(\omega \right)$. The dipole moving at the light velocity will generate a Cherenkov-like THz radiation with the spatial distribution of its energy spectral density denoted as [18]:

(6) $$\frac{d^{2}W}{d\omega d\Omega }=\frac{{\left|j_{z}\left(\omega \right)\right|}^{2}}{4\pi \varepsilon_{0}c}\frac{\rho_{0}^{4}\sin^{2}\theta }{{\left(1-cos\theta \right)}^{2}}\sin^{2}\left[\frac{L\omega }{2c}\left(1-cos\theta \right)\right],$$

where ω is the frequency of the radiation, θ is the radiation angle with respect to the laser propagation axis, $\varepsilon_{0}$ and *c* are the dielectric constant and the speed of the light in vacuum, respectively, and $\rho_{0}$ and L are the radius and length of the filament, respectively. Based on Equation (6), considering the Fourier spectrum of the measured $E_{y,rad}^{lower}\left(L/2\right)$ as $j_{z}\left(\omega \right)$, the energy distribution of the observed radially polarized THz radiation from a longitudinally dc-biased filament can be simulated, as shown in Figure 7a. The corresponding spatial distribution of the THz amplitude and its vertical components are shown in Figure 7b and Figure 7c, respectively. The diameters of the off-axis parabolic mirrors used in the experiment were 50 mm, so we only considered the THz signals within the spatial limit of these mirrors in the simulation. The red and blue colors in the THz beam pattern in Figure 7c represent the upper and the lower halves of the vertical components of the radially polarized THz radiation possessing opposite polarities. If we introduce the mask method to this simulated beam pattern, we could obtain the THz amplitude as a function of *d* (defined in Figure 3), as the solid curve in Figure 7d. It is notable that the simulated result well agrees with the experimental observations.

## 4. Manipulation of the Radially Polarized THz Radiation

**Figure 8:**
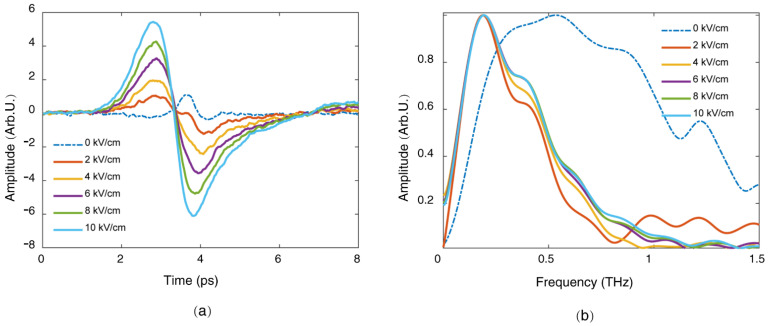
(**a**) Measured waveforms, and (**b**) corresponding spectra, of the radially polarized THz radiations when the external electric field changes from 0 to 10 kV/cm.

Based on the above mask method, the amplitude of the radially polarized THz radiation can be manipulated by adjusting the amplitude of the longitudinal external electric field. Figure 8 shows the measured waveforms and the corresponding Fourier spectra of the radially polarized THz radiation from the longitudinally dc-biased filament as the external electric field changes. When the amplitude of the external electric field increased from 2 kV/cm to 10 kV/cm, the amplitude of the radially polarized THz radiation increased linearly with respect to the amplitude of the external electric field, while the peak frequency of the THz spectrum was almost fixed around 0.2 THz. The THz peak frequency from the dc-biased filament was lower than the peak frequency of THz radiation from the filament without external electric field (0 kV/cm) because the external electric field can only influence electrons in the outer layer of the filament with a thin thickness due to the Debye shielding effect. When there is no external electric field, however, the THz signal emits from the whole filament, including the central region of the filament with relatively higher plasma density compared with the outer layer. Therefore, the frequency of the THz radiation without the external electric field was much higher, according to the relation for plasma frequency $\omega_{p}=\sqrt{\frac{n_{e}e^{2}}{m_{e}\varepsilon_{0}}}$, where $n_{e}$ is the plasma density, e is the charge of the electron, $m_{e}$ is the electron mass, and $\varepsilon_{0}$ is the dielectric constant in vacuum, respectively [11].

**Figure 9:**
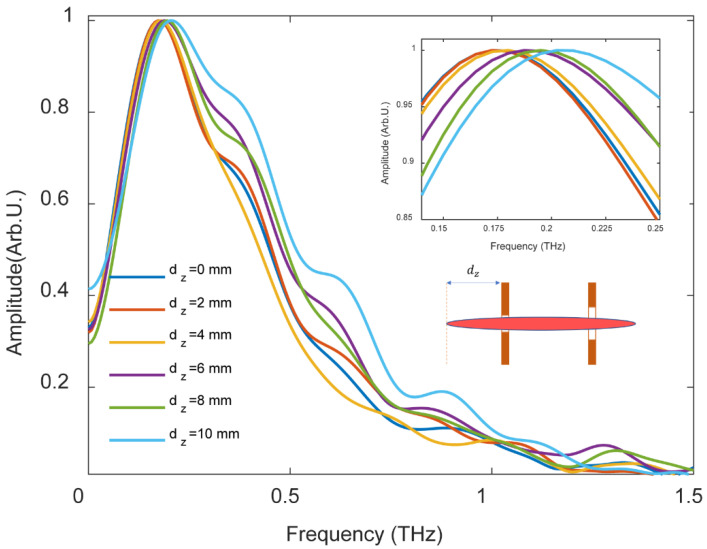
Normalized spectra of radially polarized THz radiation from a dc-biased filament when the location of the electrodes moves from the beginning to the tailing of the filament. The external electric field is 10 kV/cm. The distance from the beginning of the filament to the electrodes is defined as *d_z_*, as shown in the figure.

With this longitudinally dc-biased filament, we could also slightly manipulate the peak frequency of the radially polarized THz radiation, except for its amplitude. In a long plasma filament, the plasma density along the filament is not homogeneous. We extended the length of the filament to 2 cm by increasing the laser energy to 4 mJ. Figure 9 shows the spectra of the radially polarized THz radiation when moving the electrodes longitudinally along the filament. The THz spectrum marked with *d_z_* = 0 mm was measured when the location of the left piece of the electrodes was at the beginning of the filament, while the THz spectrum marked with *d_z_* = 10 mm was measured when the location of the electrodes was changed by 10 mm towards the tailing of the filament. It was notable that the radially polarized THz radiation from the beginning of the filament was comparably lower than that from the tailing of the filament. This could be interpreted by the fact that the plasma density was a little bit higher at the location of the geometric focus. By this technique, we could smoothly tune the peak frequency of the radially polarized THz signal from 0.17 THz to 0.21 THz.

## 5. Conclusions

We demonstrated the diagnosis of the radially polarized THz radiation from a longitudinal dc-biased laser plasma filament by introducing a movable metal mask. The amplitude and spectrum of the radially polarized THz beam was measured with a <110>-cut ZnTe crystal by modulating the THz beam pattern with the mask. Meanwhile, the linearly polarized components of the THz radiation from this dc-biased filament could be well distinguished from the THz pulses withradial polarization. The measured 1-D spatial distribution of the radially polarized THz radiation matched well with the simulation, according to the transition-Cherenkov model. Based on the mask method, the amplitude, as well as the spectrum of the radially polarized THz radiation, was manipulated by adjusting the amplitude and location of the external electric field, respectively. This work provides a new method of simultaneously measuring radially polarized and linearly polarized THz radiation, which has wide relevance in THz applications.

## Data Availability

The data presented in this study are available on request from the corresponding author.
